# K_ATP_ Channels Mediate Differential Metabolic Responses to Glucose Shortage of the Dorsomedial and Ventrolateral Oscillators in the Central Clock

**DOI:** 10.1038/s41598-017-00699-3

**Published:** 2017-04-04

**Authors:** Jyh-Jeen Yang, Ruo-Ciao Cheng, Pi-Cheng Cheng, Yi-Chi Wang, Rong-Chi Huang

**Affiliations:** 1 Department of Physiology and Pharmacology, College of Medicine, Chang Gung University, Tao-Yuan, 33305 Taiwan; 2 Healthy Aging Research Center, Chang Gung University, Tao-Yuan, 33305 Taiwan; 3 Neuroscience Research Center, Chang Gung Memorial Hospital, Linkou Medical Center, Tao-Yuan, 33305 Taiwan

## Abstract

The suprachiasmatic nucleus (SCN) central clock comprises two coupled oscillators, with light entraining the retinorecipient vasoactive intestinal peptide (VIP)-positive ventrolateral oscillator, which then entrains the arginine vasopressin (AVP)-positive dorsomedial oscillator. While glucose availability is known to alter photic entrainment, it is unclear how the SCN neurones respond to metabolic regulation and whether the two oscillators respond differently. Here we show that the ATP-sensitive K^+^ (K_ATP_) channel mediates differential responses to glucose shortage of the two oscillators. RT-PCR and electrophysiological results suggested the presence of Kir6.2/SUR1 K_ATP_ channels in the SCN neurones. Immunostaining revealed preferential distribution of Kir6.2 in the dorsomedial subregion and selective colocalization with AVP. Whole cell recordings with ATP-free pipette solution indicated larger tolbutamide-induced depolarisation and tolbutamide-sensitive conductance in dorsal SCN (dSCN) than ventral SCN (vSCN) neurones. Tolbutamide-sensitive conductance was low under perforated patch conditions but markedly enhanced by cyanide inhibition of mitochondrial respiration. Glucoprivation produced a larger steady-state inhibition in dSCN than vSCN neurones, and importantly hypoglycemia via opening K_ATP_ channels selectively inhibited the K_ATP_-expressing neurones. Our results suggest that the AVP-SCN oscillator may act as a glucose sensor to respond to glucose shortage while sparing the VIP-SCN oscillator to remain in synch with external light-dark cycle.

## Introduction

The circadian clock in the hypothalamic suprachiasmatic nucleus (SCN) is entrained by photic input to synchronize daily rhythms of physiological, metabolic, and behavioral activities in mammals. In rats, photic information from the retina is transmitted by the glutamatergic retinohypothalamic tract to reach the VIP-positive ventrolateral “core”^1, 2^, which then innervates the AVP-positive dorsomedial “shell”^3^. The anatomy is consistent with a model of two mutually but asymmetrically coupled oscillators that light entrains the retinorecipient ventrolateral oscillator, which in turn entrains a second dorsomedial oscillator (see ref. 4). Non-photic cues can also entrain the SCN clock via serotonergic projections from the median raphe nucleus and via neuropeptide Y (NPY)-ergic projections from the intergeniculate leaflet, with both pathways also targeting the ventrolateral SCN^5^. The SCN exhibits in-phase circadian rhythms in spontaneous firing rate and 2-deoxyglucose uptake, with both being higher during the day (see ref. 6). Recent data indicates that metabolic cues such as the availability of glucose can act on the SCN to alter its circadian phase and photic entrainment^7, 8^. Nevertheless, it is mostly unknown how the SCN neurones respond to metabolic regulation and whether the metabolic responses differ between dorsomedial and ventrolateral oscillators.

The ATP-sensitive K^+^ (K_ATP_) channel is a hetero-octameric complex composed of the pore-forming channel subunit (Kir6.1 or Kir6.2) and the regulatory sulfonylurea subunit (SUR1 or SUR2)^9, 10^. The K_ATP_ channel is inhibited by ATP binding to the cytoplasmic domain of the Kir6.2 subunit to stabilize the closed states of the channel and thus may act as metabolic sensor to couple cellular energetics to electrical excitability^11^. In particular, the Kir6.2-containing K_ATP_ channel plays a pivotal role in glucose homeostasis, including regulating secretion of insulin and glucagon, respectively, from pancreatic β-cells and α-cells, regulating glucose uptake in skeletal muscle, and contributing to central control of hepatic glucose output and appetite^12, 13^.

In a previous study we investigated whether the SCN neurones are sensitive to metabolic perturbation. We found that cyanide inhibition of mitochondrial respiration blocked Na/K pumps to increase intracellular Na^+^ in all SCN neurones and that a one-minute application of cyanide excited most, but not all, SCN neurones, with a subset of cells inhibited by cyanide^14^. Here we tested the idea that K_ATP_ channels mediate cyanide-induced inhibition of SCN neurones. Furthermore, results from studying forced desynchronized rats reveal that the ventrolateral and dorsomedial oscillators may become dissociated from each other to drive distinct rhythms such as locomotor activity, melatonin release, core body temperature, and sleep rhythms^4, 15–17^, suggesting that the two SCN oscillators can independently drive distinct physiological rhythms^18^. Here we investigated whether the two oscillators may differentially express K_ATP_ channels to respond differently to metabolic stresses induced by cyanide inhibition of mitochondrial respiration, glucoprivation, and hypoglycemia.

RT-PCR was used to determine the expression of mRNAs for K_ATP_ subunits and immunohistochemistry/immunofluorescence to determine the distribution pattern of Kir6.2 immunoreactivity and its colocalization with neuropeptides in the SCN. Our results indicate preferential expression of Kir6.2 in the AVP-containing dorsomedial SCN and selective colocalization of Kir6.2 with AVP. Consistently, the dSCN neurones exhibit larger tolbutamide-induced depolarisation and tolbutamide-sensitive conductance under whole cell conditions without ATP in pipette solution. Results with perforated patch recordings reveal a low resting tolbutamide-sensitive K_ATP_ conductance, which can be markedly enhanced by cyanide inhibition of mitochondrial respiration. Cell-attached recordings indicate that glucoprivation opens K_ATP_ channels to produce a larger inhibition in dSCN than vSCN neurones. Most importantly, hypoglycemia via opening K_ATP_ channels selectively inhibits the K_ATP_-expressing, mostly the dSCN, neurones, suggesting that the AVP-SCN oscillator may act as a glucose sensor to respond to glucose shortage while leaving the VIP-SCN oscillator unaltered to respond to external light-dark cycle.

## Results

### Kir6.2/SUR1 combination of ATP-sensitive K^+^ channels in the rat SCN neurones

**Figure 1:**
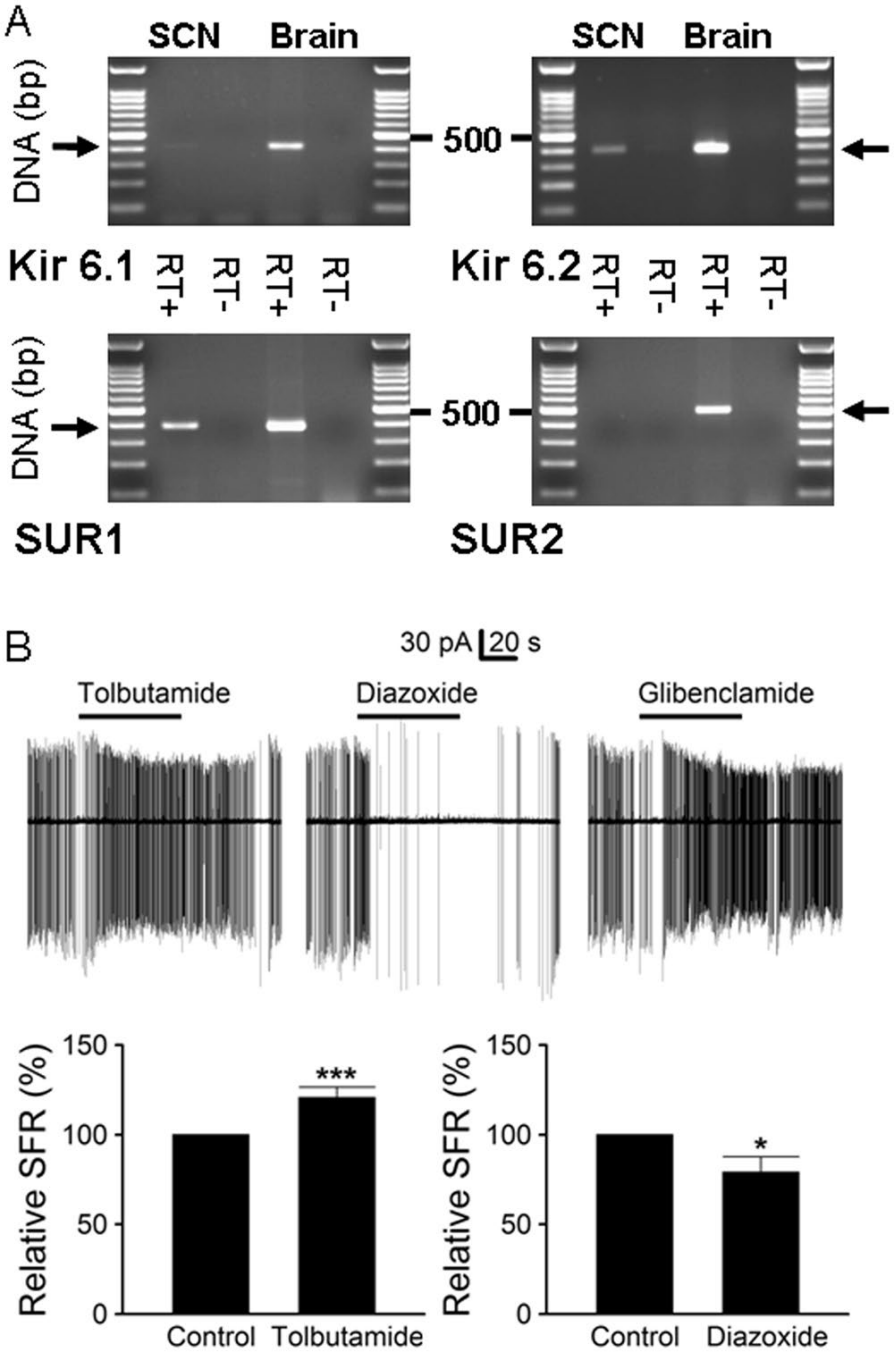
Kir6.2/SUR1 combination of K_ATP_ channels in the SCN neurones. (**A**) RT-PCR analysis indicating the expression of mRNA for pore-forming Kir6.2 and sulfonylurea subunits 1 in the SCN. Positive controls were performed using cDNA from rat brain. The expected PCR product sizes for Kir6.1, Kir6.2, SUR1, and SUR2 were 411, 385, 388, and 501 bp, respectively. Negative controls were performed using RT products with omission of reverse transcriptase (RT-) to examine the contamination of genomic DNA. (**B**) Cell-attached recordings showing the effects of the sulfonylurea on spontaneous firing of the SCN neurones (ZT 4–15). Firing responses of a representative cell to 200 μM tolbutamide (top left panel), 200 μM diazoxide (top middle panel), and 0.1 μM glibenclamide (top right panel). Note the lack of recovery of spontaneous firing after washout of glibenclamide. Daytime recordings (ZT 7). Bottom left panel: summary of experiments showing a moderate increase in firing rate by tolbutamide. Baseline spontaneous firing rate: 2.7 ± 0.3 Hz (*n* = 50). Bottom right panel: summary of experiments showing a decrease in firing rate by diazoxide. Baseline spontaneous firing rate: 3.0 ± 0.3 Hz (*n* = 17). *P < 0.05, ***P < 0.001.

RT-PCR was used to determine the expression of the channel-forming Kir6.x and sulfonylurea receptor subunits in the SCN (Fig. 1A). Positive control reactions were performed using cDNA of rat brain (Kir6.1, Kir6.2, SUR1, and SUR2) to determine the primer efficiency and anneal temperature. These primers were then used to examine the gene transcription of Kir6.x and SUR subunits in the SCN. The RT-PCR of SCN showed positive signals with primers of Kir6.2 and SUR1 as compared with the RT- control, suggesting a composition of Kir6.2/SUR1 for K_ATP_ channels in the SCN.

As the Kir6.2/SUR1 K_ATP_ channel is selectively inhibited by the sulfonylurea tolbutamide, irreversibly inhibited by glibenclamide, and opened by diazoxide^10, 19^, we used the cell-attached recording technique to study the effects of these drugs on the spontaneous firing of SCN neurones (Fig. 1B). The top panels compare the effects of 200 µM tolbutamide, 200 µM diazoxide, and 0.1 µM glibenclamide on a representative SCN neurone. The result shows a reversible increase in spontaneous firing by tolbutamide (top left panel), suggesting tonic activation of K_ATP_ channels in this particular cell. On average, the relative firing rate in tolbutamide to that in control was 121 ± 6% (*n* = 50; *P* < 0.001, paired *t*-test) (bottom left panel).

Unlike a reversible increase in the firing rate by tolbutamide, glibenclamide increased it in an irreversible manner (top right panel). The glibenclamide-induced irreversible increase in spontaneous firing is apparently due to its virtually irreversible block of pancreatic β cell-type of K_ATP_ channels (Kir6.2/SUR1)^19^. By contrast, diazoxide reduced the firing rate (top middle panel). On average, the relative firing rate in diazoxide to that in control was 79 ± 9% (*n* = 17; *P* < 0.05, paired *t*-test) (bottom right panel). Together the results indicate that tolbutamide reversibly increased, glibenclamide irreversibly increased, and diazoxide reduced the spontaneous firing of SCN neurones. The pharmacological profile of the sulfonylurea effect on spontaneous firing suggests the presence of Kir6.2/SUR1 K_ATP_ channels in SCN neurones, in accordance with the expression of mRNAs for Kir6.2 and SUR1 in the SCN (Fig. 1A). The irreversible effect of glibenclamide on firing rate in rat SCN neurones, however, is contrary to a reversible block of K_ATP_ channel by glibenclamide in dissociated hamster SCN neurones^20^.

### Selective expression of Kir6.2 immunoreactivity in the AVP-SCN neurones

**Figure 2:**
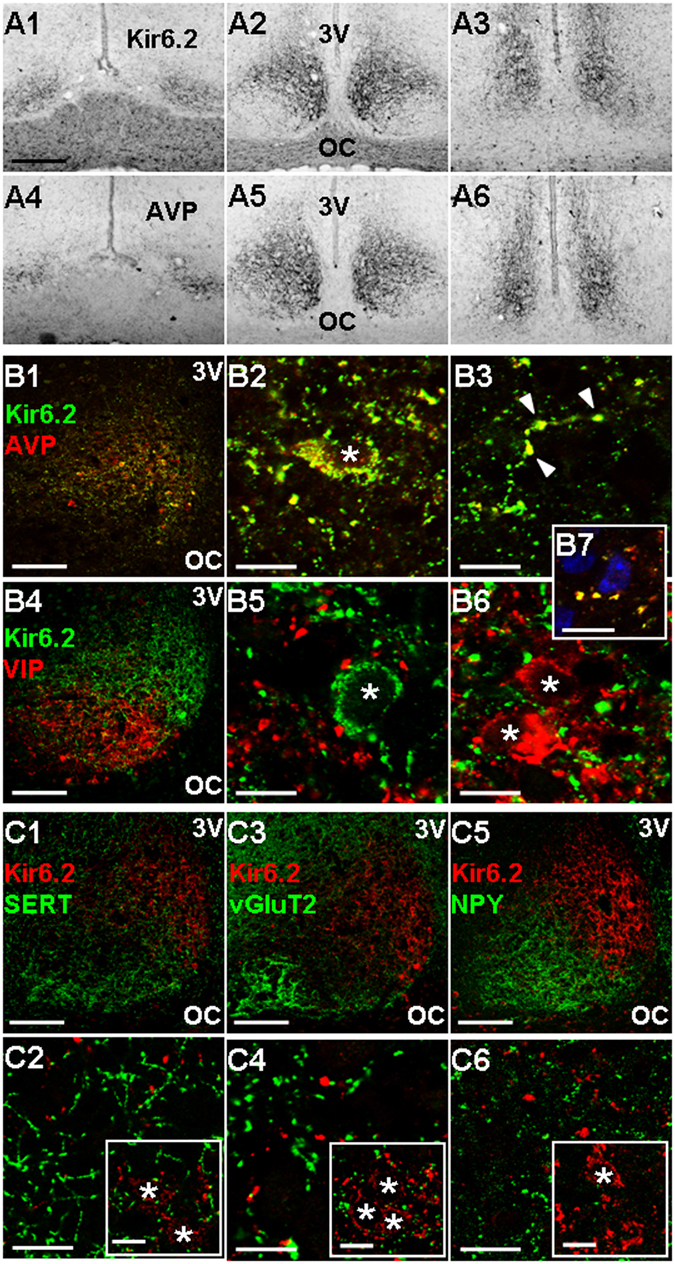
Distribution and localization of Kir6.2 immunoreactivity (ir) in the SCN. (**A**) Distribution of the Kir6.2 (A1–3) and AVP (A4-6) immunoreactivity in the rostral (A1, A4), middle (A2, A5), and caudal (A3, A6) sections of the SCN. Scale bar: 200 µm. (**B**) Selective colocalization of Kir6.2-ir with AVP-ir (B1–3) but not with VIP-ir (B4–6). Note the colocalization of Kir6.2-ir and AVP-ir in and around the soma (B2) and in varicosities along the process (marked by arrowheads, B3). Note the reciprocal apposition of VIP-ir bouton-like swellings against a Kir6.2-ir soma (B5) and Kir6.2-ir bouton-like swellings against VIP-ir somata (B6). Note also bouton-like swellings (yellow) double-stained with Kir6.2 (green) and AVP (red) apposing Hoechst-stained cells (blue) in the ventrolateral region of the SCN (B7). Scale bar: 100 µm (B1, B4); 10 µm (B2, B3, B5–7). (**C**) Lack of colocalization of Kir6.2-ir with markers for three afferent inputs SERT-ir (C1, C2), vGluT2-ir (C3, C4), NPY-ir (C5, C6). Insets: co-distribution of SERT-ir, vGluT2-ir, and NPY-ir with Kir6.2-stained somata in the ventromedial region of the mid-SCN section. Scale bar: 100 µm (C1, C3, C5); 10 µm (C2, C4, C6, insets). OC: optic chiasm. 3 V: third ventricle. Asterisks mark Hoechst-stained nuclei.

We next used a Kir6.2-specific antibody to study the distribution pattern and localization of Kir6.2 subunit in the SCN (Fig. 2). The low magnification images show the expression of Kir6.2 immunoreactivity throughout the rostrocaudal axis (Fig. 2A1–3). The distribution pattern of Kir6.2 immunoreactivity was similar to that of AVP (Fig. 2A4–6). Kir6.2-immunoreactive perikarya were confined to the dorsomedial aspect of the SCN, where immunoreactive fibres formed a dense plexus and passed out into the subparaventricular zone (not shown). The immunoreactive fibres can also be seen in the ventrolateral region where the retinorecipient neurones are located. Comparison of relative optical density between ZT 8 and ZT 14 indicates a lack of day-night variation in immunoreactivity intensity for Kir6.2 (not shown).

Double staining immunofluorescence for Kir6.2 and AVP (Fig. 2B1–3) as well as Kir6.2 and VIP (Fig. 2B4–6) were performed in the mid-SCN sections to determine the localization of Kir6.2 subunit in specific type of cells. In agreement with similar distribution pattern of Kir6.2 and AVP, there was a high degree of colocalization of AVP with Kir6.2 in and around the soma (Fig. 2B2) and in varicosities along the process (marked by arrowheads, Fig. 2B3). The AVP-partner neurophysin 2 was also found to colocalize with Kir6.2 (not shown). In contrast, no colocalization of Kir6.2 and VIP was found (Fig. 2B4–6). Instead, VIP-immunoreactive bouton-like swellings could be found to appose the Kir6.2-stained soma (Fig. 2B5), and Kir6.2-immunoreactive bouton-like swellings to appose the VIP neurones (Fig. 2B6). Similar bouton-like swellings (yellow) double-stained with Kir6.2 (green) and AVP (red) could also be found to appose Hoechst-stained, presumably VIP- and/or gastrin releasing peptide (GRP)-containing, cells (blue) located in the ventrolateral SCN (Fig. 2B7). Furthermore, there was also no colocalization between Kir6.2 and GRP or somatostatin (not shown)

To determine the possible presence of Kir6.2 in afferent inputs to the SCN, antibodies for the vesicular glutamate transporter (vGluT2), serotonin transporter (SERT), or neuropeptide Y (NPY) were used to perform double staining with Kir6.2 (Fig. 2C1,C3,C5). None of the three markers for afferent inputs were found to colocalize with Kir6.2 (Fig. 2C2,C4,C6). Nevertheless, punctate staining with anti-vGluT2 or anti-SERT could be detected to exhibit close apposition with Kir6.2-stained cells in the ventromedial SCN (insets, Fig. 2C2,C4). Taken together, the results indicate a selective expression of Kir6.2 subunits in the AVP-positive neurones.

### Preferential expression of K_ATP_ channels in the dorsal SCN (dSCN) neurones

**Figure 3:**
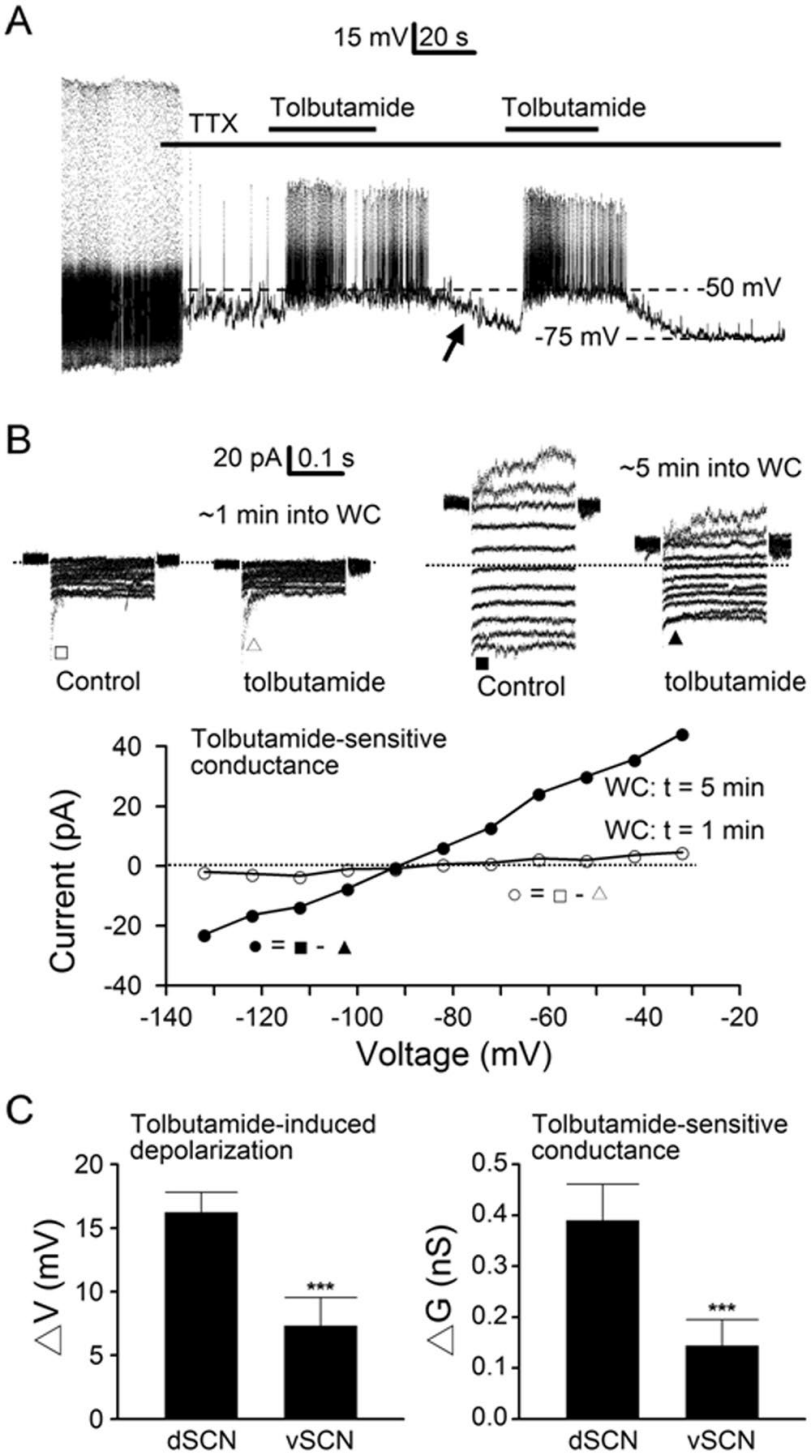
Tolbutamide effects on membrane potential and conductance under whole cell conditions with ATP-free pipette solution. (**A**) A representative dSCN neurone showing voltage responses to tolbutamide during the first few minutes into whole cell recordings. Note the much larger depolarisation by tolbutamide following spontaneous hyperpolarisation (marked by arrow), indicating the activation of K_ATP_ channels. Nighttime recordings (ZT 14). (**B**) A representative dSCN neurone showing the current responses to tolbutamide recorded at ~1 and ~5 min after breaking into whole cell (WC) recordings (top panels). Note the large leakage currents and their marked blockade by tolbutamide at ~5 min into WC (top right two panels). Bottom panel: comparison of the I-V relations of tolbutamide-sensitive conductance recorded at ~1 (○) and ~5 min (●) into WC. Daytime recordings (ZT 8). (**C**) Summary of tolbutamide-induced depolarisation (left panel) and tolbutamide-sensitive conductance (right panel), both being larger in dSCN than vSCN neurones. Baseline resting potential after ~3–5 min into WC: dSCN (−68 ± 3 mV; *n* = 17), vSCN (−64 ± 4 mV; *n* = 12). Baseline resting conductance after ~3–5 min into WC: dSCN (0.85 ± 0.11 nS; *n* = 23), vSCN (0.64 ± 0.10 nS; *n* = 22). Dotted lines are zero current levels. ***P < 0.0001.

The selective colocalization of Kir6.2 with AVP, but not VIP, would suggest a larger K_ATP_ conductance in neurones recorded from the dorsal, AVP-containing region than from the ventral, VIP-containing region of the medial SCN. To test this idea, we used the whole cell recording with ATP-free pipette solution to determine the activation of K_ATP_ channels in the SCN neurones (Fig. 3). For the experiments, we compared the voltage (Fig. 3A) and current (Fig. 3B) responses to tolbutamide before and after runup of K_ATP_ conductance after breaking into the whole-cell configuration. Figure 3A shows such a result recorded from a dSCN neurone from the dorsal region of the SCN. TTX (0.3 µM) was added to block the Na^+^-dependent action potentials to better determine the resting membrane potential. Note that this particular cell fired Ca^2+^ spikes at a very low rate in TTX. Tolbutamide depolarised the cell to ~−50 mV to fire higher frequency of Ca^2+^ spikes, followed by spontaneous hyperpolarisation (marked by arrow). A second application of tolbutamide again depolarised the cell to ~−50 mV to fire another bout of Ca^2+^ spikes. The result suggests that the spontaneous hyperpolarisation is mostly due to the runup of K_ATP_ conductance. As the spontaneous hyperpolarisation, if any, mostly completed within the first one to three minutes on breaking into the whole cell condition, the values we used for statistics were those obtained after three minutes into the whole cell mode. On average, tolbutamide depolarised the membrane potential of dSCN neurones by 16.2 ± 1.6 mV (*n* = 17), approximately twice larger than an average of 7.3 ± 2.2 mV (*n* = 12; *P* < 0.0001, Student’s *t*-test) in vSCN neurones (Fig. 3C, left panel).

Figure 3B shows the determination of K_ATP_ conductance with tolbutamide in a representative dSCN neurone. For the experiment, the membrane currents were activated by holding the cell at −52 mV (after correction of −12 mV junction potential), and voltage pulses were stepped to potentials between −32 and −132 mV in 10-mV decrements. The tolbutamide-sensitive conductance was determined right after breaking into the whole cell (WC) condition (top left two panels) and again after spontaneous runup of resting conductance (top right two panels). Bottom panel shows the I-V relations, constructed by plotting against the membrane potentials the leakage current amplitude averaged over the duration of 20–50 ms after the capacitative transient (marked by □, △, ■, and ▲), of tolbutamide-sensitive conductance before (~1 min into WC; open circles) and after (~5 min into WC; filled circles) runup. The result indicates a large increase in tolbutamide-sensitive conductance, from 0.09 nS before to 0.68 nS after runup. Similar results were also obtained by studying the effect of tolbutamide on cells recorded first in perforated patch condition and then after breaking into the whole cell condition (not shown). On average, tolbutamide blocked the resting conductance by 0.39 ± 0.07 nS (*n* = 23) in dSCN neurones, approximately twice larger than an average of 0.14 ± 0.05 nS (*n* = 22; *P* < 0.0001, Student’s *t*-test) in vSCN neurones (Fig. 3C, right panel). To determine whether there was temporal variation in tolbutamide effects, cells were grouped according to the time they were recorded during the day (ZT 4–12) or at night (ZT 12–19). The result indicates no day-night variation in the tolbutamide-sensitive conductance for dSCN (0.40 ± 0.09 nS (*n* = 13) versus 0.37 ± 0.12 nS (*n* = 10); *P* > 0.05, Student’s *t*-test) or vSCN neurones (0.14 ± 0.16 nS (*n* = 15) versus 0.15 ± 0.11 nS (*n* = 7); *P* > 0.05, Student’s *t*-test).

### Tonic activation of K_ATP_ channels is low in the SCN neurones

**Figure 4:**
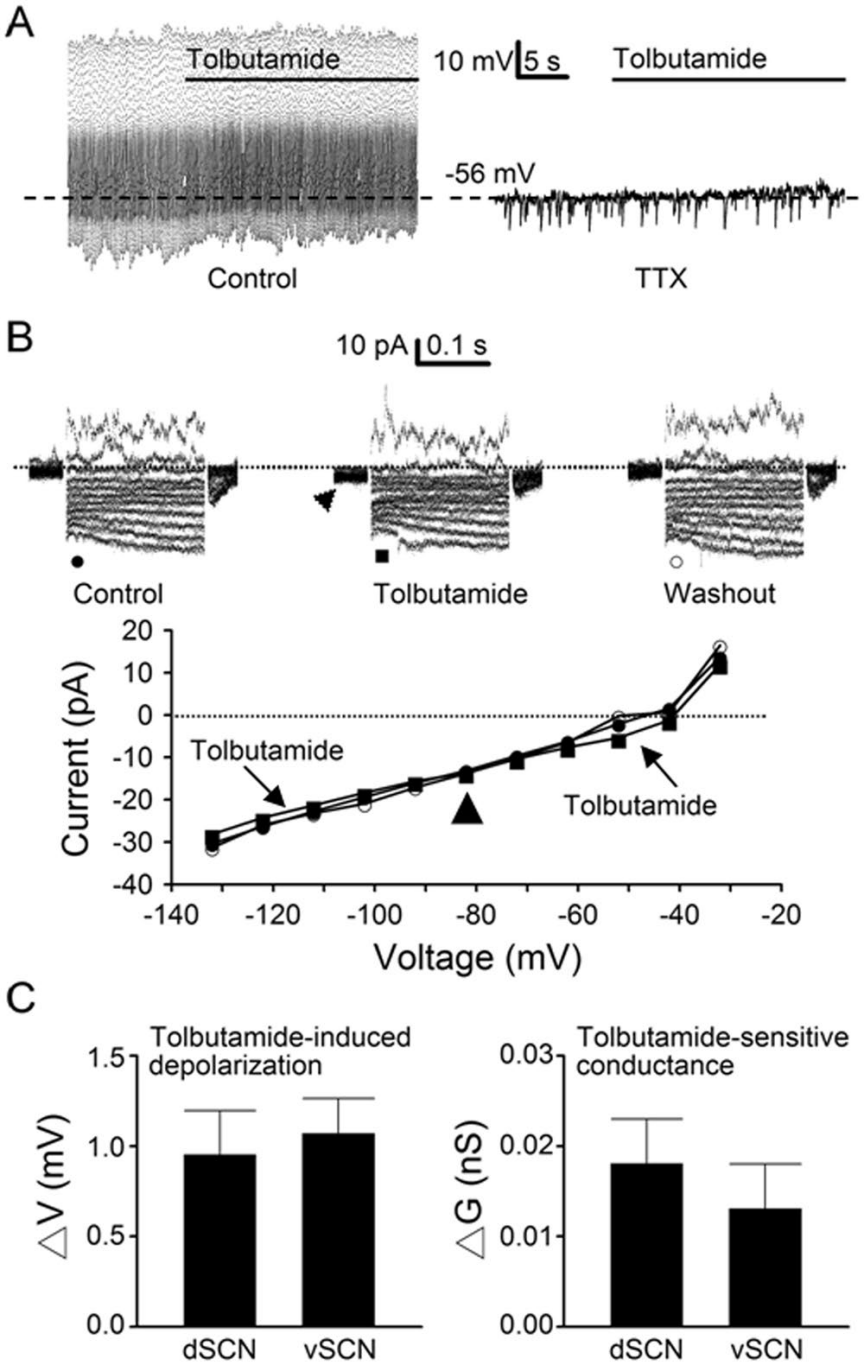
Tolbutamide effects on membrane potential and conductance with perforated patch recordings. (**A**) A representative dSCN neurone showing the effects of tolbutamide on spontaneous firing (left panel) and membrane potential (right panel) after the addition of TTX to block action potentials. Note the weak depolarisation by tolbutamide. Daytime recordings (ZT 7). (**B**) A representative dSCN neurone showing the reversible effects of tolbutamide on the membrane current (top panels) and the I-V relations (bottom panel). Note the small inward current induced by tolbutamide at a holding potential of −52 mV (marked by arrowhead). Nighttime recordings (ZT 13). (**C**) Summary of tolbutamide-induced depolarisation (left panel) and tolbutamide-sensitive conductance (right panel). Baseline resting potential: dSCN (−56 ± 1 mV; *n* = 39), vSCN (−56 ± 1 mV; *n* = 39). Baseline resting conductance: dSCN (0.38 ± 0.05 nS; *n* = 17), vSCN (0.31 ± 0.04 nS; *n* = 17). Dotted lines are zero current levels.

To determine the level of tonic activation of K_ATP_ channels under resting conditions, perforated patch recordings were used to investigate the effects of tolbutamide on the resting membrane potential and conductance (Fig. 4). For measuring the resting membrane potential, 0.3 µM TTX was added to block Na^+^-dependent action potentials. Figure 4A shows the firing and voltage responses of a representative dSCN neurone to tolbutamide in control (left panel) and after blocking the Na^+^-dependent action potentials with TTX (right panel), respectively. On average, tolbutamide depolarised the resting membrane potential by 0.9 ± 0.2 mV (*n* = 39) in dSCN neurones and 1.1 ± 0.2 mV (*n* = 39; *P* > 0.05, Student’s *t*-test) in vSCN neurones (Fig. 4C, left panel).

Figure 4B shows the effect of tolbutamide on the membrane conductance of a representative dSCN neurone that was depolarised by tolbutamide by 3.5 mV in current-clamped recordings. Top panels show the reversible effects of tolbutamide on the membrane currents. Consistent with its depolarising effect, tolbutamide produced a small inward shift in the holding currents at −52 mV (marked by arrowhead, top middle panel). Bottom panel shows the I-V relations constructed by plotting against the membrane potentials the leakage current amplitude averaged over the duration of 20–50 ms after the capacitative transient before (●), during (■), and after (○) tolbutamide. Tolbutamide reversibly blocked a leakage current which reversed at −82 mV (marked by arrowhead), close to the predicted K^+^ equilibrium potential of −87 mV. On average, the tolbutamide-sensitive resting conductance was 0.018 ± 0.005 nS (*n* = 17) in dSCN neurones and 0.013 ± 0.005 nS (*n* = 17; *P* > 0.05, Student’s *t*-test) in vSCN neurones (Fig. 4C, right panel).

To determine whether there was temporal variation in tolbutamide effects, cells were grouped according to the time they were recorded during the day (ZT 4–12) or at night (ZT 12–19). The results also indicate a possible lack of day-night variation in tolbutamide-induced depolarisation in either dSCN or vSCN neurones. The tolbutamide-induced depolarisation recorded during the day and at night averaged, respectively, 0.7 ± 0.4 mV (*n* = 19) and 1.2 ± 0.3 mV (*n* = 20; *P* > 0.05, Student’s *t*-test) in dSCN neurones, and 1.3 ± 0.3 mV (*n* = 23) and 0.8 ± 0.3 mV (*n* = 16; *P* > 0.05, Student’s *t*-test) in vSCN neurones.

### Mitochondrial inhibition activates the K_ATP_ channels in the SCN neurones

**Figure 5:**
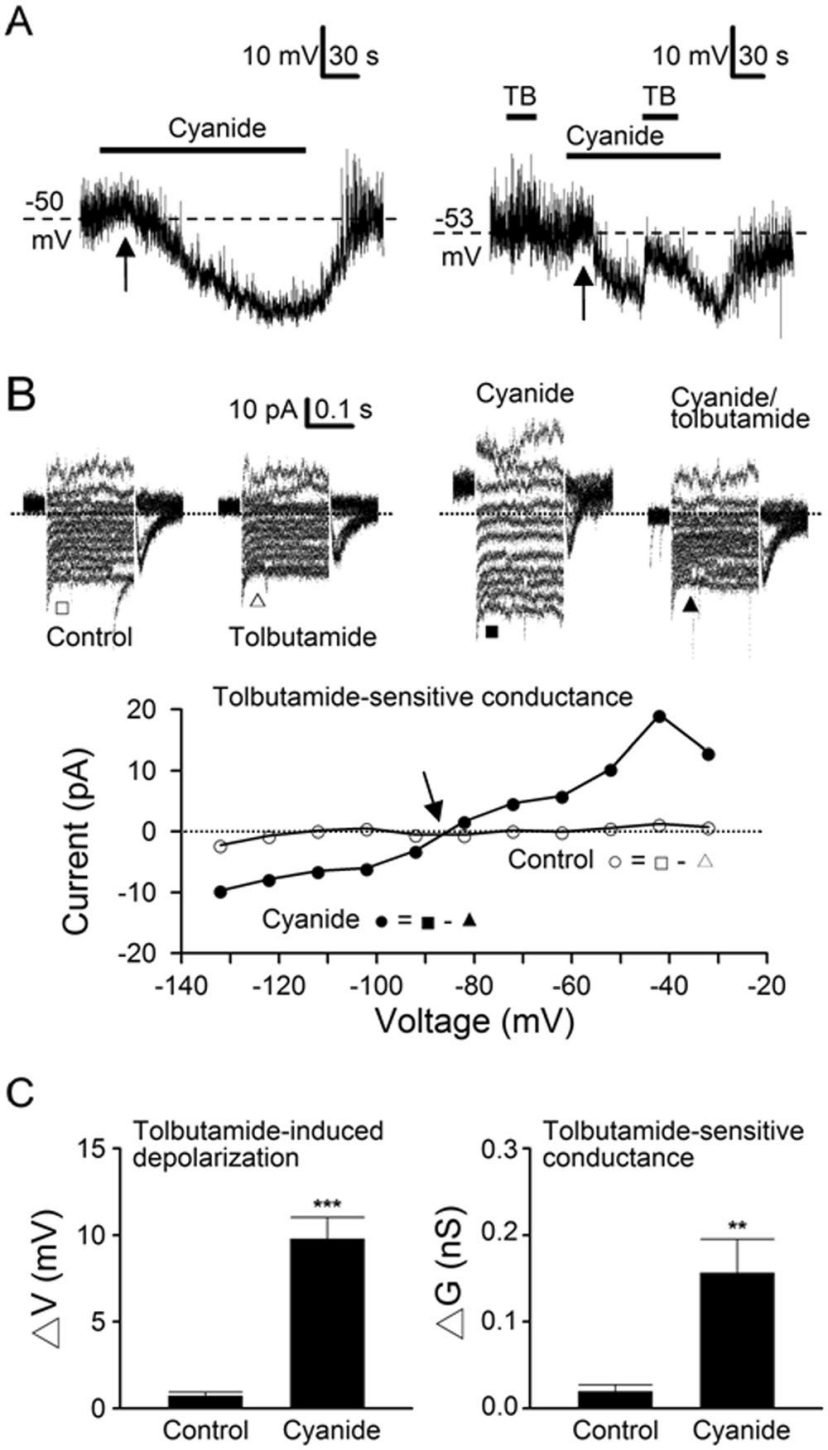
Cyanide inhibition of mitochondrial respiration activates K_ATP_ channels. (**A**) A representative dSCN neurone showing the voltage responses to cyanide (left panel), and then again the tolbutamide (TB) effects on the membrane potential in control and in the presence of cyanide (right panel). Note the early depolarisations (marked by arrows) followed by tolbutamide-sensitive hyperpolarisation. Daytime recordings (ZT 4). (**B**) A representative dSCN neurone showing tolbutamide effects on the membrane currents in control (top left two panels) and then in the presence of cyanide (top right two panels). Bottom panel: the I-V relations of tolbutamide-sensitive conductance in control (○) and in the presence of cyanide (●). Note the marked increase in the tolbutamide-sensitive conductance by cyanide. Daytime recordings (ZT 5). (**C**) Summary of tolbutamide-induced depolarisation (left panel) and tolbutamide-sensitive conductance (right panel) in control and in the presence of cyanide. TTX was present to block the action potentials. Baseline resting potential: −55 ± 2 mV (*n* = 16). Baseline resting conductance: 0.32 ± 0.03 nS (*n* = 16). Dotted lines are zero current levels. **P < 0.005, ***P < 0.0001.

The tonic activation of K_ATP_ conductance in perforated patch conditions is of low level in either dSCN (0.018 nS, ~5% of 0.39 nS in whole cell conditions) or vSCN (0.013 nS, ~10% of 0.14 nS in whole cell conditions), suggesting that K_ATP_ is mostly closed in physiological conditions, most likely by basal levels of ATP. To test this idea, we selected cells with hyperpolarising response to cyanide and compared the tolbutamide effects before and during the application of cyanide as exemplified by a representative dSCN neurone shown in Fig. 5A. For this particular cell, 1 mM NaCN initially depolarised the resting membrane potential cell by 3 mV (from −50 to −47 mV, marked by arrow) and then gradually hyperpolarised it by −19 mV (from −50 mV to −69 mV) in ~3 min (left panel). The cyanide-induced early depolarisation has been shown to be mediated by its blockade of Na/K pumps^14^. The second application of NaCN again induced early depolarisation (marked by arrow) followed by hyperpolarisation, which was nearly completely reversed by the addition of tolbutamide (TB; right panel). For a total of 16 cells, tolbutamide depolarisation averaged 0.7 ± 0.3 mV (*n* = 16) in control and 9.8 ± 1.3 mV (*n* = 16; *P* < 0.0001, paired *t*-test) during cyanide hyperpolarisation (Fig. 5C, left panel). Comparison of tolbutamide effects during cyanide-induced hyperpolarisation indicates a slight larger depolarisation at day (11.9 ± 1.8 mV; *n* = 8) than at night (7.7 ± 1.5 mV; *n* = 8; *P* = 0.09, Student’s *t*-test), but not of statistical significance.

To determine the K_ATP_ conductance activated by cyanide, we compared the tolbutamide effects on membrane currents in the absence and then the presence of cyanide (Fig. 5B). For this particular cell, tolbutamide had negligible effect on membrane currents in control (top left two panels), and the tolbutamide-sensitive conductance (○; bottom panel) was small (0.02 nS). Cyanide markedly enhanced membrane currents, which were mostly blocked by tolbutamide (top right two panels), and the tolbutamide-sensitive conductance (●; bottom panel) has increased 10 fold to 0.24 nS. The tolbutamide-sensitive conductance in cyanide intersects with that in control at a potential close to the predicted K^+^ equilibrium potential of −87 mV (marked by arrow, bottom panel). On average, tolbutamide-sensitive conductance increased from 0.02 ± 0.01 nS (*n* = 16) in control to 0.16 ± 0.04 nS (*n* = 16; *P* < 0.005, paired *t*-test) in cyanide (Fig. 5C, right panel). Comparison of tolbutamide-sensitive conductance in cyanide also indicates a slight larger conductance at day (0.19 ± 0.06 nS; *n* = 10) than at night (0.10 ± 0.03 nS; *n* = 6; *P* = 0.24, Student’s *t*-test), but not of statistical significance.

### Glucoprivation produces biphasic effect on spontaneous firing

**Figure 6:**
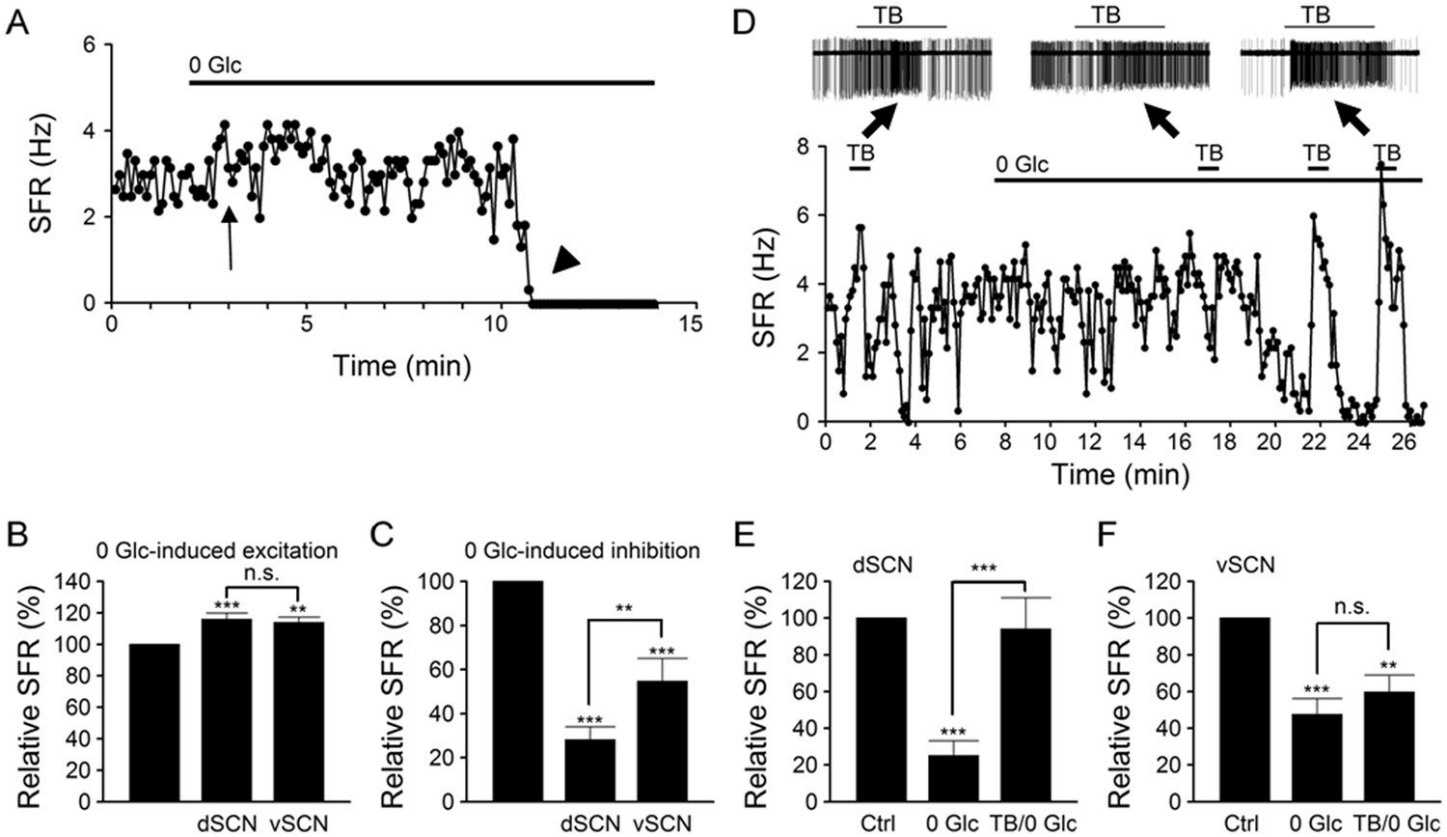
Glucoprivation produces biphasic effect on spontaneous firing. (**A**) A representative dSCN neurone showing the biphasic effects, early excitation (marked by arrow) and delayed inhibition (marked by arrowhead), of glucose-free (0 Glc) solution on spontaneous firing rate (SFR). Daytime recordings (ZT 8). (**B**) Statistics showing that glucoprivation provoked similar early excitation in dSCN and vSCN neurones. (**C**) Statistics showing that glucoprivation produced a larger steady-state inhibition in dSCN than vSCN neurones. Baseline spontaneous firing rate: dSCN (3.4 ± 0.4 Hz; *n* = 18), vSCN (3.5 ± 0.3 Hz; *n* = 12). (**D**) A representative dSCN neurone showing that tolbutamide-sensitive K_ATP_ channels mediate glucoprivation-induced inhibition of spontaneous firing. Insets show the original traces. Note the marked excitatory effect of tolbutamide (TB) (right inset) when glucose-free solution began to inhibit spontaneous firing. Nighttime recordings (ZT 16). (**E**,**F**) Statistics showing the relative spontaneous firing rate in control (Ctrl), steady-state inhibition by glucose-free solution (0 Glc), and the effect of tolbutamide (TB/0 Glc) in dSCN (**E**) and vSCN (**F**) neurones. Baseline spontaneous firing rate: dSCN (3.0 ± 0.3 Hz; *n* = 12), vSCN (3.2 ± 0.4 Hz; *n* = 9). **P < 0.01, ***P < 0.001.

The activation of K_ATP_ channels by mitochondrial inhibition with cyanide suggests that glucoprivation and hypoglycemia may compromise respiration to open the K_ATP_ channels. To test this idea, we first determined the effects of glucoprivation (0 Glc) on the spontaneous firing in the SCN neurones (Fig. 6). Only cells with spontaneous firing rate higher than 2 Hz were selected for experiments for better resolution and statistics purpose. Results from cell-attached recordings indicate that 0 Glc in general produced a biphasic, excitatory followed by inhibitory, effect on spontaneous firing, as exemplified by a representative dSCN neurone shown in Fig. 6A. As indicated, the early excitation (marked by arrow) was of small or moderate magnitude and normally occurred in the first few minutes following the withdrawal of glucose. In contrast, the delayed inhibition was marked, even to the point of total suppression of spontaneous firing (marked by arrowhead).

To quantify the effect of 0 Glc on spontaneous firing, relative firing rate was calculated by taking the ratio of spontaneous firing rate in glucose-free solution to that in control. Glucose-free solution increased the firing rate during the first few minutes in both dSCN and vSCN neurones, with the relative firing rate being 116 ± 4% (*n* = 18 cells; *P* < 0.001, ANOVA) and 114 ± 4% (*n* = 12 cells; *P* < 0.01, ANOVA), respectively (Fig. 6B). The results indicate that glucoprivation induces a similar early excitation in both dSCN and vSCN neurones (*P* > 0.05, ANOVA).

In contrast, although nearly all SCN neurones (29 out of 30 cells) were eventually inhibited by glucose withdrawal for a duration up to 30 min, the extent of inhibition differs between dSCN and vSCN neurones. The relative firing rate at steady state inhibition (normally between 10 and 30 min after glucose withdrawal) averaged 28 ± 6% (*n* = 18 cells; *P* < 0.001, ANOVA) and 55 ± 10% (*n* = 12 cells; *P* < 0.001, ANOVA) for the dSCN and vSCN neurones, respectively (Fig. 6C). The results indicate that glucoprivation induces a stronger inhibition of firing in dSCN than vSCN neurones (*P* < 0.01, ANOVA).

To determine whether the activation of K_ATP_ channels mediates the differential inhibition of dSCN and vSCN neurones by glucoprivation, we investigated the effects of tolbutamide on spontaneous firing in control and during 0 Glc-induced inhibition of firing. Figure 6D shows such a result from a representative dSCN neurone. As indicated, tolbutamide had a small effect on spontaneous firing in control and in 0 Glc when the firing rate was not much altered, but markedly increase the firing when 0 Glc had nearly completely suppressed the firing. The result indicates that for this dSCN neurone, 0 Glc-induced inhibition of spontaneous firing was mediated by opening of the K_ATP_ channels. Figure 6E summarizes the statistics. For a total of 12 dSCN neurones, 0 Glc reduced the firing rate to 25 ± 8% of control (*n* = 12 cells; *P* < 0.001, ANOVA), which was then returned to 94 ± 17% by tolbutamide (*n* = 12 cells; *P* > 0.05, ANOVA). The result suggests that glucoprivation activated K_ATP_ channels to inhibit dSCN neurones. In contrast, for most (7/9) vSCN neurones, tolbutamide had negligible effect during 0 Glc-induced steady state inhibition of firing. For a total of 9 cells, 0 Glc reduced the firing rate to 48 ± 9% (*n* = 9 cells; *P* < 0.001, ANOVA), which was insignificantly increased to 60 ± 9% by tolbutamide (*n* = 9 cells; *P* < 0.01, ANOVA) (Fig. 6F). The result suggests mechanisms other than activation of K_ATP_ channels in mediating glucoprivation-induced inhibition of vSCN neurones.

### Hypoglycemia activates K_ATP_ channels to mediate delayed firing inhibition

**Figure 7:**
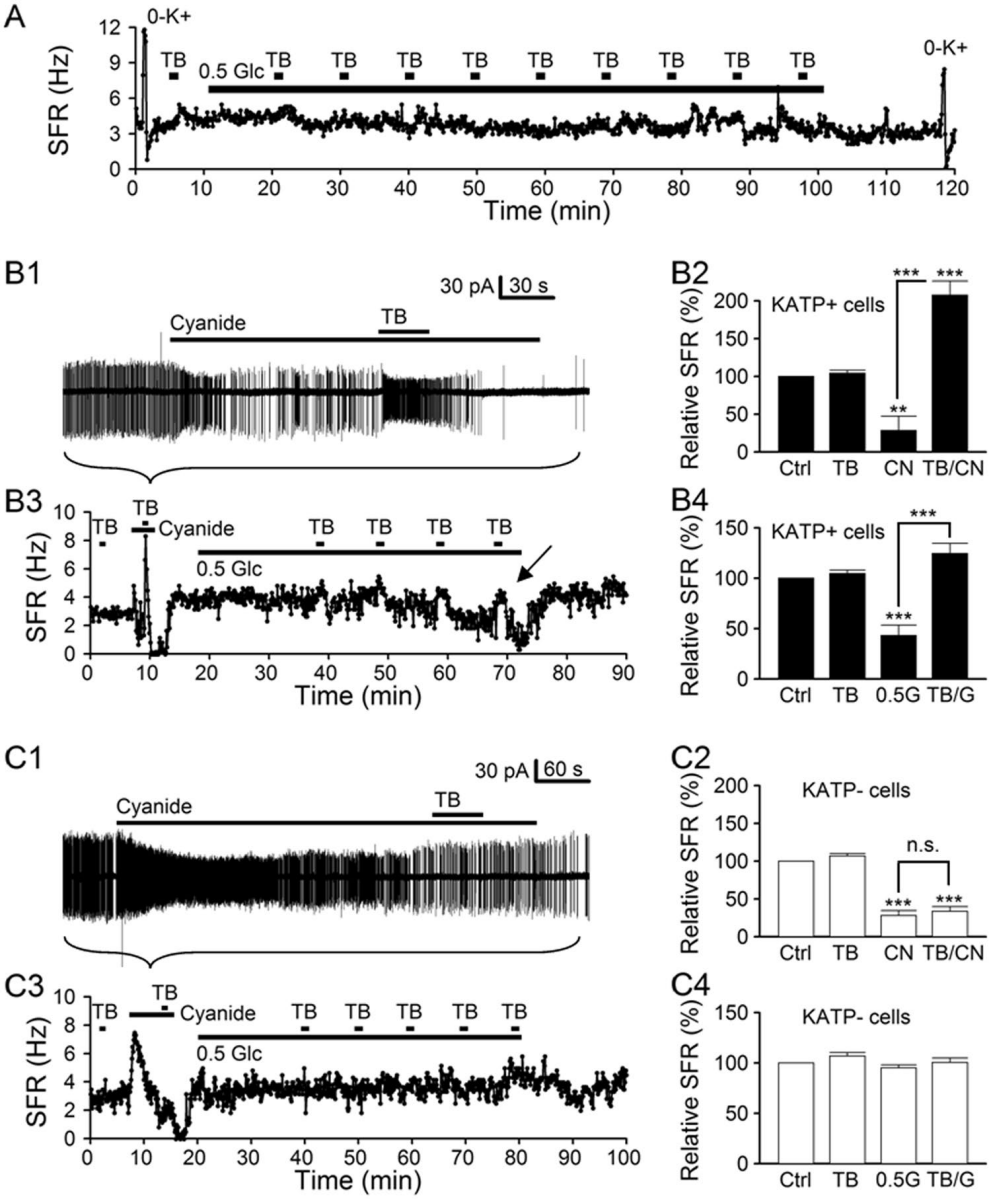
Hypoglycemia selectively inhibits the K_ATP_-expressing SCN neurones. (**A**) A representative dSCN neurone showing a negligible response to the application of hypoglycemic (0.5 mM glucose) solution for 90 min. K^+^-free (0-K^+^) solution was applied to evaluate the cell condition (see Results for details). Daytime recordings (ZT 8). (**B**) Firing responses of K_ATP_-expressing (KATP+) cells to cyanide (B1,B2) and to 0.5 mM glucose solution (B3,B4). For this representative KATP+ cell, the addition of cyanide rapidly increased and then decreased the firing rate (B1), with tolbutamide (TB) having little effect on firing rate in control (Ctrl) but greatly increasing it during cyanide inhibition (B1,B3). Daytime recordings (ZT 7). Statistics showing that cyanide (CN, B2) or hypoglycemia (0.5 G, B4) inhibits KATP + cells by opening tolbutamide-sensitive K_ATP_ channels (TB/CN, B2; TB/G, B4). Baseline spontaneous firing rate in KATP + cells: 3.2 ± 0.4 Hz (*n* = 11). (**C**) Firing responses of KATP– cells to cyanide (**C1,C2**) and to 0.5 mM glucose solution (C3,C4). For this representative KATP– cell, the addition of cyanide rapidly increased but then slowly decreased the firing rate (C1), with tolbutamide (TB) having little effect on firing rate in control and during cyanide inhibition (C1,C3). Nighttime recordings (ZT 14). Statistics showing that cyanide inhibits KATP– cells by mechanisms other than opening tolbutamide-sensitive K_ATP_ channels (C2) and that hypoglycemia (0.5 G) had no effect on KATP– cells for up to 1 hr (C4). Baseline spontaneous firing rate in KATP– cells: 3.6 ± 0.2 Hz (*n* = 9). **P < 0.01, ***P < 0.001.

Glucose shortage (induced by insulin administration or 30 hr fasting) is known to attenuate light-induced phase shifts in mice^21^. Previous studies indicate a lowering of glucose concentration to 0.2 mM with insulin-induced hypoglycemia^22^ and to 0.7 mM after overnight fasting^23^. Here we investigated the effects of hypoglycemia (0.5 mM glucose) on the firing responses of SCN neurones. As a longer time was expected for 0.5 mM glucose solution to take effect than for glucose-free solution, we also monitored the cell condition by determining the firing responses to K^+^-free (0-K^+^) solution, which blocks the Na/K pump to depolarise the membrane potential and increase spontaneous firing of SCN neurones^14, 24^. Figure 7A shows one such result recorded from a representative dSCN neurone. Note the increase in spontaneous firing in response to K^+^-free solution, both before and after hypoglycemia treatment, suggesting a good cell condition. For this particular neurone, however, lowering extracellular glucose to 0.5 mM for up to 90 min had minimal effect on the firing, nor did the application of tolbutamide before and during the application of 0.5 mM glucose solution. The result may suggest a lack of expression of K_ATP_ channels so that hypoglycemia had little effect on the excitability for up to ~90 min. Alternatively, it could be that this particular neurone did express K_ATP_ channels but an exposure duration of 90 min was simply not long enough to activate these channels to inhibit spontaneous firing.

To avoid the interpretation ambiguity, the following experiments were performed by first determining the presence of K_ATP_ channels (by adding 1 mM NaCN and then tolbutamide), followed by the application of 0.5 mM glucose solution for 1 hr to determine whether hypoglycemia can activate K_ATP_ channels within this period of time (Fig. 7B3,C3). Our results indicate that, among a total of 20 (11 dSCN + 9 vSCN) neurones, 11 (9 dSCN + 2 vSCN) expressed K_ATP_ channels as determined by their responses to cyanide and tolbutamide, and 8 (6 dSCN + 2 vSCN) of the 11 cells had their firing rate suppressed by the opening of K_ATP_ channels in response to hypoglycemia for up to 1 hr. In other words, 1 hr exposure to the hypoglycemic solution is able to open K_ATP_ channels in 73% (8 out of 11) K_ATP_-expressing SCN neurones, but not long enough for the rest (27%; 3 out of 11). Nevertheless, 0.5 mM glucose did not appear to increase spontaneous firing in either dSCN (102 ± 2%; *n* = 11 cells; *P* > 0.05) or vSCN (103 ± 3%; *n* = 9 cells; *P* > 0.05) neurones.

Figure 7B1,B3 show the effect of hypoglycemia on the firing response of a representative K_ATP_-expressing dSCN neurone. Note the cyanide-induced initial increase and then decrease in the firing in less than 1 min, reminiscent of cyanide-induced depolarisation and hyperpolarisation shown in Fig. 5A. Tolbutamide dramatically increased spontaneous firing during cyanide-induced inhibition but not in control condition, suggesting that cyanide inhibits spontaneous firing by activating tolbutamide-sensitive K_ATP_ channels. On average, the firing rate was suppressed by 1 mM NaCN to 29 ± 19% (*n* = 11 cells) and was then significantly increased by tolbutamide to 207 ± 19% (*n* = 11 cells) (Fig. 7B2). Figure 7B3 plots the time course of change in spontaneous firing rate, which began to decrease ~40 min after applying 0.5 mM glucose solution. Importantly, tolbutamide reversed hypoglycemia-induced firing inhibition (marked by arrow), suggesting hypoglycemia-induced activation of K_ATP_ channels. On average, the firing rate was reduced by 1-hr hypoglycemic treatment to 43 ± 10% (*n* = 11 cells) and was then significantly increased by tolbutamide to 125 ± 10% (*n* = 11 cells) (Fig. 7B4).

Figure 7C1,C3 show the effect of hypoglycemia on the firing response of a representative vSCN neurone that does not express K_ATP_ channels. Note that cyanide also produced a biphasic effect on spontaneous firing, but the inhibition developed more slowly over a course of several minutes (cf. Fig. 7B1,B3). Furthermore, tolbutamide did not alter the firing rate during cyanide-induced firing inhibition, suggesting that tolbutamide-sensitive K_ATP_ channels contribute minimally to the inhibition. On average, the firing rate was suppressed by 1 mM NaCN to 28 ± 7% (*n* = 9 cells) and was insignificantly increased by tolbutamide to 34 ± 6% (*n* = 9 cells) (Fig. 7C2). Figure 7C3 plots the time course of change in spontaneous firing rate, which was little affected by 1-hr application of 0.5 mM glucose solution. Tolbutamide also had no apparent effect on the firing rate. On average, the firing rate was insignificantly reduced by 1-hr hypoglycemic treatment to 95 ± 3% (*n* = 9 cells) and was also not significantly increased by tolbutamide to 100 ± 5% (*n* = 9 cells) (Fig. 7C4). Taken together, the results indicate that hypoglycemia via opening K_ATP_ channels selectively inhibits K_ATP_-expressing SCN (mostly dSCN) neurones.

## Discussion

This study demonstrates a critical role of K_ATP_ channels in mediating differential responses to glucose shortage in the (AVP-containing) dorsomedial and (VIP-containing) ventrolateral oscillators of the rat SCN. Specifically, hypoglycemia via opening K_ATP_ channels selectively inhibits K_ATP_-expressing SCN (mostly dSCN) neurones. The intense colocalization of Kir6.2 with AVP in punctate staining in and around somata and in bouton-like swellings along fibres within and out of the SCN suggests functional involvement of K_ATP_ channels in the regulation of AVP release. The results suggest that hypoglycemic regulation of the circadian clock may involve disjointed metabolic responses of the AVP-SCN and VIP-SCN oscillators and, perhaps, altered circadian output and network integration via way of AVP release.

The SCN express mRNAs for Kir6.2 and SUR1 and the effects of the sulfonylurea on the spontaneous firing of SCN neurones accord with the pharmacological profile of Kir6.2/SUR1 K_ATP_ channel^10, 19^. Immunohistochemical localization of Kir6.2 indicates a preferential distribution of Kir6.2 immunoreactivity in the dorsomedial SCN, with no day-night variation in the immunoreactivity between ZT8 and ZT14, and double immunofluorescence staining indicates selective colocalization of Kir6.2 with AVP. Specifically, high levels of Kir6.2 and AVP colocalization are found in punctate forms in somatodendritic areas and in bouton-like swellings along the fibres and around somata within the SCN and projecting upward to the subparaventricular zone, suggesting the possible involvement of Kir6.2 in the regulation of AVP release (see later).

Consistent with the selective localization of Kir6.2 in the AVP-SCN neurones and lack of day-night variation in the intensity of Kir6.2 immunoreactivity, the magnitudes of tolbutamide-induced depolarisation and tolbutamide-sensitive conductance, as determined with ATP-free pipette solution in whole cell conditions, are both larger in the dSCN neurones and do not vary between day and night.

The level of tonic activation of K_ATP_ conductance (~0.02 nS) is small, as demonstrated with perforated patch recordings, ~10% of that (0.24 nS) recorded with whole cell recordings, and contributes very slightly (~−1.0 mV) to the resting membrane potential. Together with a possible lack of day-night variation in the tolbutamide-induced depolarisation, it appears that the K_ATP_ channels play at best a small role in regulating membrane excitability under resting conditions and may not contribute to the diurnal rhythm of neuronal excitability.

The low level of tonic activation of K_ATP_ conductance suggests that K_ATP_ is mostly closed in physiological conditions, most likely by basal levels of ATP. Indeed, cyanide inhibition of mitochondrial ATP synthesis markedly enhances the K_ATP_ conductance (by ~10 times) to hyperpolarise the SCN neurones. The result suggests a role of K_ATP_ channels in coupling energetic status with neuronal excitability in K_ATP_-expressing SCN neurones.

The activation of K_ATP_ channels by cyanide inhibition of mitochondrial ATP synthesis suggests that glucoprivation and hypoglycemia may activate K_ATP_ channels to preferentially inhibit dSCN neurones. Indeed, our results reveal that hypoglycemia via opening K_ATP_ channels selectively inhibit SCN neurones that express K_ATP_ channels. Specifically, 1-hr exposure to hypoglycemia inhibits 73% of cells that express K_ATP_ channels, but has no effect on cells that do not. Presumably, a longer time (>1 hr) of exposure to hypoglycemia should also inhibit the rest of 27% cells that express K_ATP_ channels. The presence of K_ATP_ channels in 82% of dSCN neurones versus 22% of vSCN neurones suggests that hypoglycemia should inhibit approximately four times more dSCN than vSCN neurones. Nevertheless, as the Kir6.2 immunoreactivity is found to colocalize only with the AVP immunoreactivity, it is very likely that the K_ATP_-expressing vSCN neurones may in fact be the AVP-SCN neurones.

Contrary to the selective inhibition of K_ATP_-expressing neurones by hypoglycemia, glucoprivation (for up to 30 min) inhibits almost all (97%) SCN neurones tested, but with larger steady-state inhibition of firing in dSCN than in vSCN neurones. Furthermore, tolbutamide reverses glucoprivation-induced firing inhibition in dSCN, but not vSCN, neurones, suggesting that K_ATP_ channels mediate glucoprivation-induced inhibition in dSCN, but not vSCN, neurones. It remains to be determined the mechanisms underlying glucoprivation-induced inhibition in the vSCN neurones that do not express K_ATP_ channels. Preliminary results suggest a lack of involvement of Ca^2+^-dependent K^+^ channels in mediating such inhibition.

Glucoprivation also increases neuronal firing rate in the first few minutes in both dSCN and vSCN neurones. The glucoprivation-induced early excitation, seemingly a slower and smaller version of cyanide-induced initial excitation, is likely mediated by metabolic inhibition of Na/K pumps to increase spontaneous firing in all SCN neurones^14^. In contrast, there is no apparent increase in spontaneous firing during exposure to hypoglycemia. In other words, hypoglycemia appears to selectively compromise mitochondrial respiration associated to K_ATP_ channels but not to Na/K pumps. How this functional compartmentation is achieved remains to be elucidated.

The availability of glucose is known to alter photic entrainment in mice^21, 25^. Although available evidence suggests the involvement of extra-SCN regions such as the ventromedial hypothalamus in mediating metabolic regulation of photic response, a direct action on the SCN cannot be excluded^8, 26^. Our finding of hypoglycemia-induced selective inhibition of K_ATP_-expressing neurones suggests a mechanism for metabolic regulation of the SCN. In particular, the disjointed firing responses of dSCN and vSCN neurones to hypoglycemia suggest that the two oscillators could potentially desynchronize from each other at times of glucose shortage, with consequence of impaired photic entrainment. The suggestion is in line with a dual oscillator model of the SCN that the ventrolateral and dorsomedial oscillators can independently drive distinct physiological rhythms^18^, and their misalignment at the time of light stimulation impairs the ability of light to phase shift the locomotor activity^27^.

On the other hand, the selective colocalization of Kir6.2 and AVP suggests a possible involvement of K_ATP_ channels in the regulation of AVP release. Experiments with reverse microdialysis indicate that the resting release of AVP from the SCN relies on [Ca^2+^]_i_, being increased by high K^+^ solutions and reduced by blocking transmembrane Ca^2+^ influx^28^. As such, at times of glucose shortage, selective opening of K_ATP_ channels in the AVP-SCN neurones could lower neuronal firing and [Ca^2+^]_i_ to reduce AVP release, with two possible consequences.

First, morphological and functional evidence suggests an important role of the AVP-SCN neurones in mediating intercellular communication between dorsomedial and ventrolateral oscillators^29–33^. In this study, bouton-like swellings double-stained with Kir6.2 and AVP were also found to closely appose the Hoechst-stained, presumably VIP- and/or GRP-containing, cell located in the ventrolateral SCN. Hypoglycemia could potentially reduce AVP signaling in the ventrolateral SCN to affect light-induced entrainment. In this context, it is worth mentioning that reduced responsiveness to light-induced phase delay has been recently reported in mice with selective knockout of *Bmal1* in the AVP-SCN neurones^33^. These mice have marked reduction in the expression of genes involved in intercellular communication including *Avp* and *Prokineticin 2*, as well as reduced responsiveness to light-induced *Per1* expression and phase delay in the locomotor activity. There is also evidence indicating that AVP via V1a receptor positively regulates the expression of *Prokineticin 2* in the SCN^34^.

Second, the AVP-SCN neurones also act as autonomic pacemaker^35^, using AVP as an inhibitory output signal for corticosterone release in the rat (see ref. 36). Thus, the reduced release of AVP at times of glucose shortage could potentially relieve SCN targets from inhibition by the SCN, for example, to increase corticosterone levels, as has been shown in food-restricted rats^37^. Interestingly, the AVP-SCN neurones also contain Prokineticin 2, which acts as a circadian clock output and may be involved in food shortage-associated thermoregulation and energy expenditure^38–41^. In this context, the AVP-SCN oscillator may act as a glucose sensor to coordinate bodily functions in response to glucose shortage while sparing the VIP-SCN oscillator to remain in synch with external light-dark cycle.

In conclusion, K_ATP_ channels mediate differential metabolic responses to glucose shortage of the AVP-SCN and VIP-SCN oscillators. The hypoglycemia-induced preferential, if not selective, inhibition of the AVP-SCN neurones may allow the AVP-SCN oscillator to differentially affect its targets to alter behaviors to adapt to times of glucose shortage while leaving the VIP-SCN oscillator unaltered to respond to external light-dark cycle.

## Methods

### Hypothalamic brain slices and reduced SCN preparations

All experiments were carried out according to procedures approved by the Institutional Animal Care and Use Committee of Chang Gung University. Sprague-Dawley rats (18–24 days old) were kept in a temperature-controlled room under a 12:12 light:dark cycle (light on 0700–1900 hr). Lights-on was designated Zeitgeber time (ZT) 0. For daytime and nighttime recordings, the animal was killed at ZT 2 and ZT 10, respectively. An animal of either sex was carefully restrained by hand to reduce stress and killed by decapitation using a small rodent guillotine without anaesthesia, and the brain was put in an ice-cold artificial cerebrospinal fluid (ACSF) prebubbled with 95% O_2_–5% CO_2_. The ACSF contained (in mM): 125 NaCl, 3.5 KCl, 2 CaCl_2_, 1.5 MgCl_2_, 26 NaHCO_3_, 1.2 NaH_2_PO_4_, 10 glucose. A coronal slice (200–300 µm) containing the SCN and the optic chiasm was cut with a DSK microslicer DTK-1000 (Ted Pella, Redding, CA, USA), and was then incubated at room temperature (22–25 °C) in the incubation solution, which contained (in mM): 140 NaCl, 3.5 KCl, 2 CaCl_2_, 1.5 MgCl_2_, 10 glucose, 10 HEPES, pH 7.4, bubbled with 100% O_2_.

The ventral or dorsal region of the SCN is defined as the upper or lower one third of the SCN, by drawing two imaginary lines parallel to the optic chiasm and dividing the SCN into three approximately equal-size divisions^24^. For electrical recordings, a reduced SCN preparation was obtained by excising a small piece of tissue (circa one-ninth the size of SCN) from the dorsal or ventral region of the medial SCN using a fine needle (Cat no. 26002-10, Fine Science Tools, Foster City, CA, USA), followed by further trimming down to 4–10 smaller pieces with a short strip of razor blade. The reduced preparation (containing tens of cells, see Fig. 1 of ref. 42) was then transferred to a coverslip precoated with poly-D-lysine (Sigma-Aldrich, St Louis, MO, USA) in a recording chamber for recording. The SCN neurones of the reduced preparation could be identified visually with an inverted microscope (Olympus IX70 and IX71, Japan). The preparation thus obtained allows rapid application of drugs^43^ and has been used to demonstrate diurnal rhythms in both spontaneous firing and Na/K pump activity^44^.

### Electrical recordings

The reduced SCN preparation was perfused with bath solution containing (in mM): 140 NaCl, 3.5 KCl, 2 CaCl_2_, 1.5 MgCl_2_, 10 glucose, 10 HEPES, pH adjusted to 7.4 with NaOH. The perfusion rate was set at 0.6 ml/min and solution change was completed in ~1 s judging from the measurement of junction potential. The patch solution contained (in mM): 20 NaCl, 1 CaCl_2_, 2 MgCl_2_, 110 K-gluconate, 11 EGTA, 10 HEPES, pH adjusted to 7.3 with KOH. Na-ATP (3 mM) was added in patch solution when needed. The measured liquid junction potential was −12 mV^45^ and was corrected for in the presentation of data obtained with whole cell and perforated patch recordings. Pipette resistance was 4~6 MΩ. For perforated patch recordings, the patch pipette also included nystatin (Sigma-Aldrich, St Louis, MO, USA) at a final concentration of 250 µg/ml prepared from a stock solution (25 mg/ml DMSO). All recordings were made with Axopatch 200B amplifier (Axon Instruments, Foster City, CA, USA) at room temperature (22–25 °C). The spontaneous firing was recorded at room temperature in the cell-attached and perforated patch current-clamped configurations. The spike counts, in 6-s epochs, always began only after stable recordings were made. At least one or two minutes of spontaneous firing rate were counted before the application of drugs. Membrane potentials and membrane currents were recorded using the whole cell and perforated patch recording techniques. The signal was low-pass filtered at 1–5 KHz and digitised on-line at 2–10 KHz via a 12-bit A/D digitising board (DT2821F-DI, Data Translation, Marboro, MA, USA) with a custom-made program written in the C Language. Data were analyzed and plotted with custom-made programs written in Visual Basic 6.0 and the commercial software GraphPad PRISM (GraphPad Software, San Diego, CA, USA). Data are given as means ± SEM and were analyzed with Student’s *t-*test or paired *t*-test or with ANOVA, followed by Tukey’s test for comparison of selected pairs.

### Drugs

Stock solutions of tolbutamide (200 mM in 100% ethanol), diazoxide (20 mM in DMSO), and glibenclamide (0.1 mM in DMSO) were stored at −20 °C, and were diluted to reach final concentrations. Sodium cyanide was directly added to the bath to achieve the final concentrations. These chemicals were purchased from Sigma-Aldrich (St Louis, MO, USA). K^+^-free solutions was prepared with omission of extracellular K^+^, glucose-free solution, omission of extracellular glucose, and 0.5 mM glucose was added to glucose-free solution to create the hypoglycemic solution. Although the glucose-free and hypoglycemic solutions (see Figs 6 and 7) were not osmo-compensated for with sucrose, the firing rate of the SCN neurons was similar in the absence or presence of sucrose to replace the omitted glucose.

### Immunohistochemistry and immunofluorescence

Sprague-Dawley rats (23–25 days old) were deeply anesthetised with Zoletil (40 mg/kg, i.p.; Virbac Laboratories, Carros, France) and fixed by transcardial perfusion with PBS and then with 4% paraformaldehyde (500 ml/animal). Brains were removed and post-fixed overnight (more than 16 hr) in 4% paraformaldehyde, followed by dehydration with 30% sucrose in PBS for another 24 hr. Twenty-micrometer-thick coronal sections through the hypothalamus region containing the SCN were cut on a cryostat (−20 °C), collected in antifreeze solution, and stored in −20 °C freezer until further processing.

For immunohistochemical staining, sections (20 µm) were treated with 0.3% H_2_O_2_ for 15 min to quench endogenous peroxidase, and then incubated overnight at 4 °C in PBS containing 2% serum, 0.3% Triton X-100, and primary antibodies against Kir6.2 (rabbit anti-Kir6.2; 1:5000; APC-020; Alomone Labs, Jerusalem, Israel)^46^ and AVP (rabbit anti-AVP; 1:3000; AB1565; Millipore, Temecula, CA, USA)^47^. Sections were then treated with goat anti-rabbit biotinylated secondary antibody for 1 hr at room temperature (22–25 °C). Sections were then rinsed in PBS and incubated in avidin-biotin complex (ABC Elite Kit, Vector Labs, Burlingame, CA, USA) for 1 hr according to the manufacturer’s instructions. After two 10-min washes in 0.1 M sodium acetate, sections were stained with diaminobenzidine. Sections were photographed and analyzed with an inverted microscope (Olympus IX71, Japan) integrated with the MT20 illumination system (Olympus Biosystems, Planegg, Germany). Immunoreactivity for Kir6.2 was quantified by calculating the relative optical density from the mid-SCN sections with ImageJ 1.43 u (NIH).

For immunofluorescence staining, sections (20 µm) were washed for 20–30 min in PBS and then incubated overnight at 4 °C in PBS containing 2% serum, 0.3% Triton X-100, and primary antibodies against Kir6.2 (rabbit anti-Kir6.2; 1:500; APC-020; Alomone Labs, Jerusalem, Israel), AVP (guinea pig anti-AVP; 1:500; T-5048; Peninsula Laboratories, San Carlos, CA, USA)^48^, VIP (guinea pig anti-VIP; 1:500; T-5030; Peninsula Laboratories, San Carlos, CA, USA)^49^, GRP (goat anti-GRP; 1:500; sc-7788; Santa Cruz, CA, USA)^49^, neurophysin 2 (goat anti-neurophysin 2; 1:500; sc-27093; Santa Cruz, CA, USA)^50^, somatostatin (mouse anti-somatostatin; 1:500; sc-74556; Santa Cruz, CA, USA)^51^, vesicular glutamate transporter type 2 (vGluT2) (guinea pig anti-vGluT2; 1:500; AB2251; Millipore, Temecula, CA, USA)^52^, serotonin transporter (SERT) (mouse anti-SERT; 1:500, MAB1564; Millipore, Temecula, CA, USA)^53^, or NPY (guinea pig anti-NPY; 1:500, AB10341; Abcam, Cambridge, MA, USA)^54^. Sections were then treated with respective Alexa Fluor secondary antibodies 488 or 568 (diluted 1:300; Molecular Probes, Eugene, OR, USA) and Hoechst 33342 (B-2261; Sigma, St. Louis, MO, USA) for 1 hr at room temperature. After rinse in PBS, sections were coverslipped with ProLong Gold anti-fade reagent (P36930; Molecular Probes, Eugene, OR, USA) and photographed with Zeiss LSM 510 confocal microscope. Contrast and brightness were optimized using Adobe Photoshop (Adobe Systems, San Jose, CA, USA).

### RT-PCR analysis of Kir6.1, Kir6.2, SUR1, and SUR2 expression

Total RNA of SCN was extracted using the Absolutely RNA Nanoprep kit (Stratagene, La Jolla, CA, USA) according to the manufacturer’s guide; total RNA of rat brain was purchased from BioChain Institute Inc (Newark, CA, USA). RNA samples were treated with DNaseI for 13–15 min at 25 °C to eliminate genomic DNA contamination. The resulting RNA was reverse-transcribed (RT) to cDNA using ReverTra Ace (TOYOBO, Osaka, Japan) with oligo(dT) primers in a total volume of 20 μl. One-tenth of RT products were used as templates (2 μl) to perform PCR reaction. RT reaction with omission of reverse transcriptase was used as templates for negative control PCR. Primers used for RT-PCR were as follows:

Kir6.1 forward 5′-TTGGGTTTGGAGGGAGAATG-3′;

Kir6.1 reverse 5′-ACAGGGGGCTACGCTTATCA-3′;

Kir6.2 forward 5′-CTGCCTTCCTTTTCTCCATC-3′;

Kir6.2 reverse 5′-TTACCACCCACACCGTTCTC-3′;

SUR1 forward 5′-TGGGGAACGGGGCATCAACT-3′;

SUR1 reverse 5′-TGGCTCTGGGGCTTTTCTC-3′;

SUR2 forward 5′-GCAAGAGCGTGGAAGAGAC-3′;

SUR2 reverse 5′-TGCCCCATGAGAAGTATCC-3′;

The thermal cycling condition of RT-PCR was 94 °C for 3 min, followed by 35 cycles of 94 °C for 30 s, 60 °C for 30 s, and 72 °C for 30 s, and then 72 °C for 7 min. PCR amplified products were electrophoresed in 1.5% agarose gels, stained with ethidium bromide, and photographed.

## References

[1] Meijer JH, Schwartz WJ (2003). In search of the pathways for light-induced pacemaker resetting in the suprachiasmatic nucleus. J. Biol. Rhythms.

[2] Golombek DA, Rosenstein RE (2010). Physiology of circadian entrainment. Physiol. Rev..

[3] Leak RK, Card JP, Moore RY (1999). Suprachiasmatic pacemaker organization analyzed by viral transynaptic transport. Brain Res..

[4] Schwartz MD (2009). Dissociation of circadian and light inhibition of melatonin release through forced desynchronization in the rat. Proc. Natl. Acad. Sci. USA.

[5] Morin LP, Allen CN (2006). The circadian visual system, 2005. Brain Res. Rev..

[6] Newman GC, Hospod FE, Patlak CS, Moore RY (1992). Analysis of *in vitro* glucose utilization in a circadian pacemaker model. J. Neurosci..

[7] Green CB, Takahashi JS, Bass J (2008). The meter of metabolism. Cell.

[8] Challet E (2010). Interactions between light, mealtime and calorie restriction to control daily timing in mammals. J. Comp. Physiol. B.

[9] Ashcroft FM, Gribble FM (1998). Correlating structure and function in ATP-sensitive K^+^ channels. Trends Neurosci..

[10] Babenko AP, Aguilar-Bryan L, Bryan J (1998). A view of SUR/K_IR_6.X, K_ATP_ channels. Annu. Rev. Physiol..

[11] Nichols CG (2006). K_ATP_ channels as molecular sensors of cellular metabolism. Nature.

[12] Miki T, Seino S (2005). Roles of KATP channels as metabolic sensors in acute metabolic changes. J. Mol. Cell. Cardiol..

[13] McTaggart JS, Clark RH, Ashcroft FM (2010). The role of the KATP channel in glucose homeostasis in health and disease: more than meets the islet. J. Physiol..

[14] Wang Y-C, Yang J-J, Huang R-C (2012). Intracellular Na^+^ and metabolic modulation of Na/K pump and excitability in the rat suprachiasmatic nucleus neurons. J. Neurophysiol..

[15] de la Iglesia HO, Cambras T, Schwartz WJ, Diez-Noguera A (2004). Forced desynchronization of dual circadian oscillators within the rat suprachiasmatic nucleus. Curr. Biol..

[16] Cambras T (2007). Circadian desynchronization of core body temperature and sleep stages in the rat. Proc. Natl. Acad. Sci. USA.

[17] Lee ML, Swanson BE, de la Iglesia HO (2009). Circadian timing of REM sleep is coupled to an oscillator within the dorsomedial suprachiasmatic nucleus. Curr. Biol..

[18] Schwartz WJ (2009). Circadian rhythms: a tale of two nuclei. Curr. Biol..

[19] Ashfield R, Gribble FM, Ashcroft SJH, Ashcroft FM (1999). Identification of the high-affinity tolbutamide site on the SUR1 subunit of the K_ATP_ channel. Diabetes.

[20] Hall AC, Hoffmaster RM, Stern EL, Harrington ME, Bickar D (1997). Suprachiasmatic nucleus neurons are glucose sensitive. J. Biol. Rhythm.

[21] Challet E, Losee-Olson S, Turek FW (1999). Reduced glucose availability attenuates circadian responses to light in mice. Am. J. Physiol..

[22] Silver IA, Erecińska M (1994). Extracellular glucose concentration in mammalian brain: continuous monitoring of changes during increased neuronal activity and upon limitation in oxygen supply in normo-, hypo-, and hyperglycemic animals. J. Neurosci..

[23] de Vries MG, Arseneau LM, Lawson ME, Beverly JL (2003). Extracellular glucose in rat ventromedial hypothalamus during acute and recurrent hypoglycemia. Diabetes.

[24] Wang Y-C, Huang R-C (2006). Effects of sodium pump activity on spontaneous firing in neurons of the rat suprachiasmatic nucleus. J. Neurophysiol..

[25] Challet E, van Reeth O, Turek FW (1999). Altered circadian responses to light in streptozotocin-induced diabetic mice. Am. J. Physiol..

[26] Challet E, Bernard DJ, Turek FW (1999). Gold-thioglucose-induced hypothalamic lesions inhibit metabolic modulation of light-induced circadian phase shifts in mice. Brain Res..

[27] Schwartz MD, Congdon S, de la Iglesia HO (2010). Phase misalignment between suprachiasmatic neuronal sscillators impairs photic behavioral phase shifts but not photic induction of gene expression. J. Neurosci..

[28] Francl JM, Kaur G, Glass JD (2010). Roles of light and serotonin in the regulation of gastrin-releasing peptide and arginine vasopressin output in the hamster SCN circadian clock. Eur. J. Neurosci..

[29] Romijn HJ, Sluiter AA, Pool CW, Wortel J, Buijs RM (1997). Evidence from confocal fluorescence microscopy for a dense, reciprocal innervation between AVP-, somatostatin-, VIP/PHI-, GRP-, and VIP/PHI/GRP-immunoreactive neurons in the rat suprachiasmatic nucleus. Eur. J. Neurosci..

[30] Jacomy H, Burlet A, Bosler O (1999). Vasoactive intestinal peptide neurons as synaptic targets for vasopressin neurons in the suprachiasmatic nucleus. Double-label immunocytochemical demonstration in the rat. Neuroscience.

[31] Albus H, Vansteensel MJ, Michel S, Block GD, Meijer JH (2005). A GABAergic mechanism is necessary for coupling dissociable ventral and dorsal regional oscillators within the circadian clock. Curr. Biol..

[32] Maywood ES, Chesham JE, O’Brien JA, Hastings MH (2011). A diversity of paracrine signals sustains molecular circadian cycling in suprachiasmatic nucleus circuits. Proc. Natl. Acad. Sci. USA.

[33] Mieda M (2015). Cellular clocks in AVP neurons of the SCN are critical for interneuronal coupling regulating circadian behavior rhythm. Neuron.

[34] Li JD, Burton KJ, Zhang C, Hu SB, Zhou QY (2009). Vasopressin receptor V1a regulates circadian rhythms of locomotor activity and expression of clock-controlled genes in the suprachiasmatic nuclei. Am. J. Physiol. Regul. Integr. Comp. Physiol..

[35] Ueyama T (1999). Suprachiasmatic nucleus: a central autonomic clock. Nat. Neurosci..

[36] Kalsbeek A, Fliers E, Hofman MA, Swaab DF, Buijs RM (2010). Vasopressin and the output of the hypothalamic biological clock. J. Neuroendocrinol..

[37] Kalsbeek A, van Heerikhuize JJ, Wortel J, Buijs RM (1998). Restricted daytime feeding modifies suprachiasmatic nucleus vasopressin release in rats. J. Biol. Rhythms.

[38] Cheng MY (2002). Prokineticin 2 transmits the behavioural circadian rhythm of the suprachiasmatic nucleus. Nature.

[39] Li JD (2006). Attenuated circadian rhythms in mice lacking the prokineticin 2 gene. J. Neurosci..

[40] Prosser HM (2007). Prokineticin receptor 2 (Prokr2) is essential for the regulation of circadian behavior by the suprachiasmatic nuclei. Proc. Natl. Acad. Sci. USA.

[41] Zhou W, Li JD, Hu WP, Cheng MY, Zhou QY (2012). Prokineticin 2 is involved in the thermoregulation and energy expenditure. Regul Pept.

[42] Wang Y-C, Chen Y-S, Cheng R-C, Huang R-C (2015). Role of Na^+^/Ca^2+^ exchanger in Ca^2+^ homeostasis in the rat suprachiasmatic nucleus neurons. J. Neurophysiol..

[43] Chen C-H, Hsu Y-T, Chen C-C, Huang R-C (2009). Acid-sensing ion channels in neurons of the rat suprachiasmatic nucleus. J. Physiol..

[44] Wang H-Y, Huang R-C (2004). Diurnal modulation of the Na^+^/K^+^-ATPase and spontaneous firing in the rat retinorecipient clock neurons. J. Neurophysiol..

[45] Neher E (1992). Correction of liquid junction potentials in patch clamp experiments. Methods Enzymol..

[46] Lybaert P (2008). K_ATP_ channel subunits are expressed in the epididymal epithelium in several mammalian species. Biol. Reprod..

[47] Das M, Vihlen CS, Legradi G (2007). Hypothalamic and brainstem sources of pituitary adenylate cyclase-activating polypeptide nerve fibres innervating the hypothalamic paraventricular nucleus in the rat. J. Comp. Neurol..

[48] Rood BD, De Vries GJ (2011). Vasopressin innervation of the mouse (Mus musculus) brain and spinal cord. J. Comp. Neurol..

[49] Belenky MA, Yarom Y, Pickard GE (2008). Heterogeneous expression of γ-aminobutyric acid and γ-aminobutyric acid-associated receptors and transporters in the rat suprachiasmatic nucleus. J. Comp. Neurol..

[50] Koch P (2009). Expression profile of PTPIP51 in mouse brain. J. Comp. Neurol..

[51] Zou S, Somvanshi RK, Paik S, Kumar U (2015). Colocalization of cannabinoid receptor 1 with somatostatin and neuronal nitric oxide synthase in rat brain hypothalamus. J. Mol. Neurosci..

[52] Kiss J, CsáKi Á, Csaba Z, Halász B (2008). Synaptic contacts of vesicular glutamate transporter 2 fibres on chemically identified neurons of the hypothalamic suprachiasmatic nucleus of the rat. Eur. J. Neurosci..

[53] Hundahl CA, Hannibal J, Fahrenkrug J, Dewilde S, Hay-Schmidt A (2010). Neuroglobin expression in the rat suprachiasmatic nucleus: colocalization, innervation, and response to light. J. Comp. Neurol..

[54] Wu Q, Boyle MP, Palmiter RD (2009). Loss of GABAergic signaling by AgRP neurons to the parabrachial nucleus leads to starvation. Cell.

